# Emanuel (Gus) Moran FRCP FRCPsych, DPM

**DOI:** 10.1192/bjb.2018.24

**Published:** 2018-08

**Authors:** Gerald Russell

Formerly Consultant Psychiatrist, Claybury Hospital, Woodford Bridge, and Chase Farm Hospital, Enfield, UK


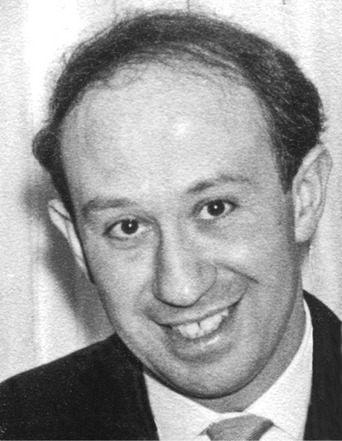


Emanuel Moran, known to many of his Maudsley colleagues (though not to family and friends) as Gus, died on 18 August 2017, aged 89. He had become the UK's foremost authority on the psychiatric and social aspects of pathological gambling. His interest had developed during his time as a psychiatric registrar at the Maudsley. He had seen patients who had attempted suicide when they were in difficulties resulting from their gambling dependence. Over the next few years, he accumulated a series of 50 such patients and published his findings in 1970 in the *British Journal of Psychiatry*.^1^ He proposed a definition of pathological gambling and put forward a tentative typology. He concluded that gambling was due to a complex interaction between personal factors and social pressures. Among the latter, he noted that the gambling urge would be triggered by new opportunities, such as the opening of a licensed betting office near the person's home or place of work.

It is striking that among the 50 subjects there were no women, a finding probably due to the limited opportunities at that time for women to indulge in serious gambling. They seldom frequented betting shops or casinos and confined themselves to bingo halls, playing that sedate game in the company of friendly neighbours. It was only in the late 1980s that fruit machines were introduced into bingo halls and there was a rapid increase in more active gambling among women. Even more pernicious was the advent of online gambling, attracting young women in particular.

A similar increase in pathological gambling occurred when regulations on gambling were relaxed. Warning signals were sounded following the 1960 Betting and Gambling Act, which had resulted in increased gambling facilities. In more recent years, Emanuel campaigned tirelessly against any further relaxation of restrictions on gambling. He was the first chairman of the Society for the Study of Gambling and insisted that no funding was received by the Society from the gambling trade. Subsequently, he founded the National Council on Gambling.

The Gambling Act’, which was passed in 2005 and became effective in 2007, had as one of its stated objectives the protection of children and other vulnerable persons from being harmed or exploited by gambling. This laudable aim has been somewhat undermined by the acceptance of an elastic definition of ‘responsible gambling’. From 1974 to 2010, Emanuel was the specialist adviser on gambling to the Royal College of Psychiatrists. Under his guidance, the College submitted crucial advice on restricting the more harmful ‘remote gambling’ (including mobile phones and the internet). He recognised that the regulation of online companies was only possible when these were based in Britain, but, in fact, most internet companies promoting gambling are now based abroad. Towards the end of his life, Emanuel felt that his efforts to reduce the dangers of gambling had been of no avail, a source of considerable distress to him. Yet his efforts had been determined and valiant.

Emanuel was born in Charlottenberg, Berlin, in 1928. His parents originally came from Kiev. His father, a Baptist minister, moved to Berlin to run a Christian mission. Emanuel was evacuated to England on his 10th birthday, where he was quite alone and knew no English, yet he pursued his schooling with ease. His parents and two younger siblings joined him in London a few months later. His further education took him to Fitzwilliam College, Cambridge, and Guy's Hospital to study medicine. After a period in neurology at the Whittington Hospital, he trained in psychiatry at the Maudsley Hospital under the influence of the formidable Professor Aubrey Lewis. From 1966 to 1980, he was a psychiatrist for Gamblers Anonymous. Through his work, gambling came to be recognised as an addictive disorder. His definition of gambling disorder was adopted by the World Health Organization. Throughout the 1970s, he worked closely with the Home Office. He advised Parliament through the Royal Commission chaired by Lord Rothschild, opposing the deregulation of gambling laws.

Emanuel was an extremely kind and considerate colleague. As a junior consultant, I had admitted a not-so-young lady who became depressed when her engagement to marry was broken. She disappeared from the ward and, justifiably, I feared the worst. The Thames River Police asked me to identify the body. Witnessing my dismay, Emanuel offered to accompany me to the mortuary, an offer I gladly accepted.

In 1965, he was appointed Consultant Psychiatrist to Claybury Hospital, Woodford Bridge, and Chase Farm Hospital, Enfield, where he worked for almost 30 years. He was clinically responsible for one of the busiest general adult sectors in north London, a role which consumed a great deal of his time and energy

In the early 1970s Emanuel began to suffer from severe ill-health, and in 1974 his wife was told that he had only weeks to live because of a bladder cancer. He survived for another 40 years, for which he felt indebted to two urosurgeons, but his illness greatly affected his subsequent quality of life. He took delight in his family. Jane was his wife for 51 years. His elder son, Paul, became a psychiatrist, and his younger son David was active in education. He leaves five grandchildren.
